# A comparative *in vitro* study of the osteogenic and adipogenic potential of human dental pulp stem cells, gingival fibroblasts and foreskin fibroblasts

**DOI:** 10.1038/s41598-018-37981-x

**Published:** 2019-02-11

**Authors:** Riccardo Monterubbianesi, Mladen Bencun, Pierfrancesco Pagella, Anna Woloszyk, Giovanna Orsini, Thimios A. Mitsiadis

**Affiliations:** 1 Orofacial Development and Regeneration, Institute of Oral Biology, Centre for Dental Medicine, University of Zurich, Zurich, Switzerland; 2 Department of Clinical Sciences and Stomatology, Polytechnic University of Marche, Ancona, Italy

## Abstract

Human teeth contain a variety of mesenchymal stem cell populations that could be used for cell-based regenerative therapies. However, the isolation and potential use of these cells in the clinics require the extraction of functional teeth, a process that may represent a significant barrier to such treatments. Fibroblasts are highly accessible and might represent a viable alternative to dental stem cells. We thus investigated and compared the *in vitro* differentiation potential of human dental pulp stem cells (hDPSCs), gingival fibroblasts (hGFs) and foreskin fibroblasts (hFFs). These cell populations were cultured in osteogenic and adipogenic differentiation media, followed by Alizarin Red S and Oil Red O staining to visualize cytodifferentiation. Quantitative Real-Time Polymerase Chain Reaction (qRT-PCR) was performed to assess the expression of markers specific for stem cells (*NANOG, OCT-4*), osteogenic (*RUNX*2*, ALP, SP7/OSX*) and adipogenic (*PPAR-γ2, LPL*) differentiation. While fibroblasts are more prone towards adipogenic differentiation, hDPSCs exhibit a higher osteogenic potential. These results indicate that although fibroblasts possess a certain mineralization capability, hDPSCs represent the most appropriate cell population for regenerative purposes involving bone and dental tissues.

## Introduction

Stem cells play an essential role in tissue homeostasis, repair and regeneration due to their high proliferation capacity, their ability to self-renew and to differentiate towards different cell lineages^1^. Stem cells reside in specific niches, which represent specialized microenvironments that maintain the ability of stem cells to self-renew and influence their fate^2^. In recent years a variety of stem cell populations have started to be used in the clinics for regenerative/repair purposes. The fundamental idea of these cell-based therapies is that stem cells administered to the injury site can differentiate into cells that are specific to the local environment, thus supporting the repair/regeneration of the damaged tissue^3^. Mesenchymal stem cells (MSCs) can give rise to cells of different mesodermal lineages such as osteoblasts, adipocytes, chondrocytes^4^, cardiomyocytes, pericytes, endothelial cells and smooth muscle cells^3^. This potential, combined with the fact that they are easily isolated, manipulated and expanded *in vitro*^5^, make them a precious tool for successful regenerative medical trials. According to the International Society for Cellular Therapy, three minimal requirements are defined for human MSCs: (1) they have to be plastic-adherent under normal culture conditions, (2) they should express CD73, CD90 and CD105, but not CD11b or CD14, CD19 or CD79α, CD45, CD34 and HLA Class II surface markers, (3) they must be able to differentiate into osteoblasts, chondroblasts and adipocytes *in vitro*^6^.

MSCs represent inhomogeneous cell populations with varying degrees of lineage commitment^7^. Adult bone marrow was the first source of MSCs identified and in more recent years other stem cell sources were discovered in a variety of tissues and organs such as periosteum, trabecular bone, adipose tissue, synovium, skeletal muscle and teeth^3^. Dental MSCs were initially isolated from the pulp of human third molars in 2000^8^ and since then MSCs were also isolated from the pulp of exfoliated deciduous teeth, apical papilla, dental follicle, periodontal ligament^9^ and periapical cysts^10^. However, these dental-derived MSC populations vary in their expression of stem cell surface markers and in their ability to differentiate into distinctive cell lineages^11^. Human dental pulp stem cells (hDPSCs) have been extensively studied over the past years and constitute very attractive candidates regarding cell-based regenerative therapies for a variety of reasons: they can be conveniently collected from extracted adult teeth without ethical concerns^8^, they possess immunosuppressive activity^12^ and can be safely cryopreserved without affecting their differentiation properties^13^. Consequently, hDPSCs may be isolated from a patient, stored and transplanted autologously at a later date, therefore making allogenic grafting and immunosuppression redundant^14^. hDPSCs are able to differentiate into odontogenic, osteogenic, chondrogenic, adipogenic, vascular, myogenic and neurogenic lineages^11,15^. Among adipose tissue, bone marrow and dental-derived MSCs, hDPSCs produce the greatest volume of mineralized matrix and therefore have greater potential for future applications in the regeneration of damaged tooth structures or bone in mandibular defects^16^.

Fibroblasts are ubiquitously distributed in connective tissues and play a major role in synthesis and secretion of extracellular matrix, as well as in inflammation, wound healing and fibrosis^17^. MSCs and fibroblasts share several common properties in terms of morphology, cell-surface marker and gene expression patterns, as well as differentiation potential^18,19^. Indeed, *in vitro* studies have shown that fibroblasts are also plastic-adherent and capable of differentiating into bone, fat and cartilage, while expressing all of the MSC surface markers^20^. Additionally, similar to MSCs, fibroblasts are able to suppress mitogenic and allogeneic lymphocyte proliferation^21^. Because of these properties, fibroblasts hold the potential for clinical application in the treatment of many diseases and constitute a very appealing alternative for regenerative applications due to their high accessibility and availability.

Therefore, in this study we assessed the differentiation potential of two human fibroblastic populations, foreskin (hFFs) and gingival (hGFs) fibroblasts, and compared them to hDPSCs by means of *in vitro* differentiation assays, complemented with expression of specific stem cell, osteogenic and adipogenic marker genes.

## Results

### *In vitro* differentiation assays

#### Osteogenic differentiation assay

**Figure 1:**
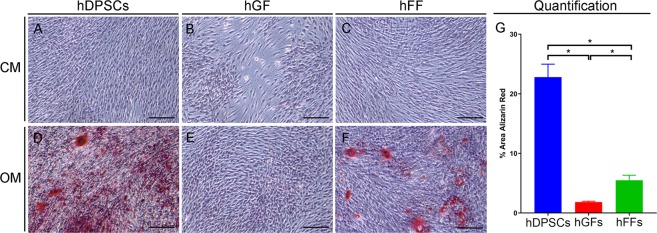
Microscopic views of Alizarin Red S staining of cultured human dental pulp stem cells (hDPSCs), human gingival fibroblasts (hGFs) and human foreskin fibroblasts (hFFs) cultured for 21 days with control medium (CM; **A**–**C**) and osteogenic medium (OM; **D**–**F**). Calcium deposits (red color) are evident in hDPSCs (**D**) and hFFs (**F**), but not in hGFs (**E**), cultured in OM. (**G**) Quantification of Alizarin Red staining as proportion of Alizarin-red-positive surface (in %). Asterisks: Mann Whitney – U/Wilcox Rank Sum Test, *p < 0.05; **p < 0.01. Colour of asterisks onto each column indicates the column used for the comparison. Abbreviations: CM, control medium; hDPSCs, human dental pulp stem cells; hFF, human foreskin fibroblasts; hGF, human gingival fibroblasts; OM, osteogenic medium. Scale bars: 100 μm.

Staining with Alizarin Red S allows visualization of extracellular calcium deposits in a bright orange-red colour. Staining revealed that hDPSCs, hGFs and hFFs cultured in control medium (CM) were not able to form mineralized nodules. When cultured for 21 days in presence of osteogenic medium (OM) hDPSCs formed a dense mineralized plexus (Fig. 1A,D). hFFs also displayed unequally distributed mineralized nodules when cultured in OM (Fig. 1C,F), whereas no mineral deposits were visible in cultures of hGFs with OM (Fig. 1B,E). Quantification of the alizarin red staining confirmed the observations, showing a significantly higher Alizarin Red-staining in hDPSCs when compared to hGFs and hFFs (Fig. 1G), as well as in hFFs compared to hGFs.

#### Adipogenic differentiation assay

**Figure 2:**
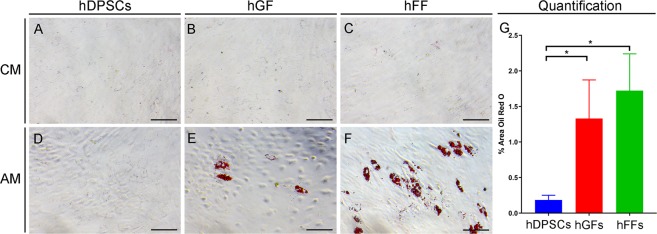
Microscopic views of Oil Red O staining of cultured hDPSCs, hGFs and hFFs on day 21 of incubation with control medium (CM; **A**–**C**) and adipogenic medium (AM; **D**–**F**). Lipid droplets in red color are visible in hGFs (**E**) and hFFs (**F**), but not in hDPSCs (**D**), cultured in the presence of AM. (**G**) Quantification of oil red O staining as proportion of oil red 0-positive surface (in %). Asterisks: Mann Whitney – U/Wilcox Rank Sum Test, *p < 0.05; **p < 0.01. Colour of asterisks onto each column indicates the column used for the comparison. Abbreviations: AM, adipogenic medium; CM, control medium; hDPSCs, human dental pulp stem cells; hFF, human foreskin fibroblasts; hGF, human gingival fibroblasts. Scale bars: 100 μm.

To monitor the adipogenic differentiation progress, cultured cell populations were dyed with Oil Red O, which stains lipid droplets in red colour. Staining revealed that after 21 days of culture in adipogenic medium (AM) hGFs and hFFs formed scattered lipid droplets (Fig. 2E,F), while no lipid droplets were observable in cultures of hDPSCs (Fig. 2D). The observation was confirmed by quantification of Oil Red O staining, which displayed significantly higher areas of Oil Red O staining in both hGFs and hFFs compared to hDPSCs (Fig. 2G). Furthermore, cells of the fibroblastic groups (Fig. 2E,F) exhibited a more spherical shape than hDPSCs (Fig. 2D).

### Gene expression analysis

The expression levels of each analysed gene within the three groups (i.e., hDPSCs, hGFs and hFFs) are shown individually after different days of incubation in osteogenic and adipogenic medium. The gene expression levels of the samples within each group (cell type) are presented relative to the gene expression level on day one of the respective group (A-C in all panels), as well as normalized for the expression of the gene in hDPSCs on day 0 (before treatment; figure D in all panels).

#### Gene expression analysis of stem cell markers

**Figure 3:**
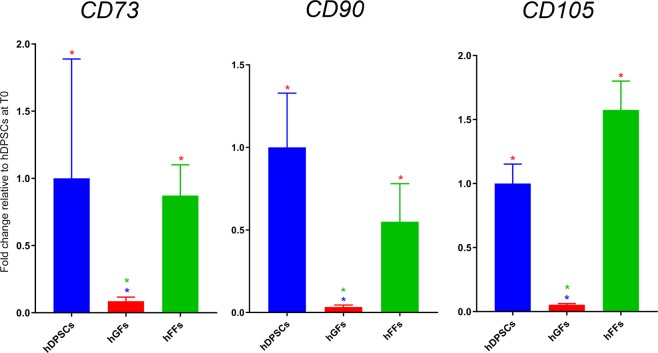
Gene expression levels of CD73, CD90 and CD105 in hDPSCs, hGFs and hFFs cultured in control conditions. Fold change is normalized to hDPSCs. Asterisks: Mann Whitney – U/Wilcox Rank Sum Test, *p < 0.05; **p < 0.01. Colour of asterisks onto each column indicates the column used for the comparison. Abbreviations: hDPSCs, human dental pulp stem cells; hFF, human foreskin fibroblasts; hGF, human gingival fibroblasts.

We first verified and quantified the expression of genes used as markers for mesenchymal stem cells, namely *CD73*, *CD90* and *CD105*^6^, in hDPSCs, hGFs and hFFs cultured in control conditions. Expression of these three genes was detected in all three cell populations analysed (Fig. 3). Of notice, hDPSCs and hFFs expressed comparable levels of *CD73*, *CD90* and *CD105*, while hGFs displayed significantly lower levels of these mRNAs (Fig. 3).

We then analysed the expression of *NANOG* and the Octamer-binding transcription factor 4 (*OCT-4*), recognized stem cell markers that play pivotal roles in the maintenance of self-renewal and pluripotency^22–27^.

**Figure 4:**
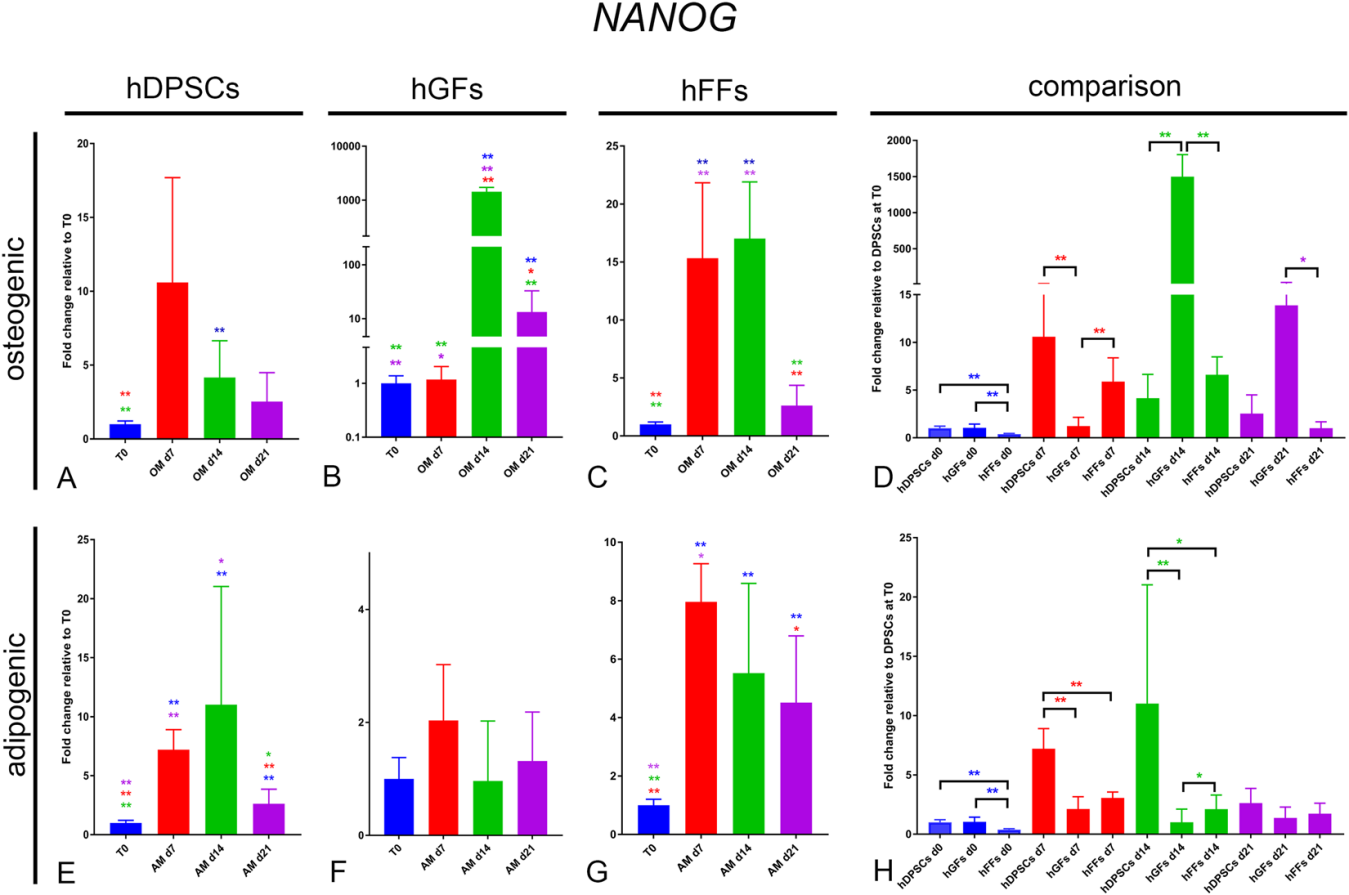
Gene expression levels of NANOG in cultured hDPSCs (**A**), hGFs (**B**) and hFFs (**C**) for 0, 7, 14 and 21 days in presence of OM and AM, relative to the respective expression levels at T0. D) Expression levels of NANOG normalized for NANOG expression in hDPSCs at T0 Asterisks: Mann Whitney – U/Wilcox Rank Sum Test, *p < 0.05; **p < 0.01. Colour of asterisks onto each column indicates the column used for the comparison. Abbreviations: hDPSCs, human dental pulp stem cells; hFF, human foreskin fibroblasts; hGF, human gingival fibroblasts; OM, osteogenic medium; AM, adipogenic medium.

NANOG: *NANOG* was surprisingly upregulated in all cell types upon treatment with differentiation media. Incubation of hDPSCs in osteogenic conditions led to a significant upregulation of the expression of *NANOG* already after one week, followed by its progressive downregulation at day 14 and 21 (Fig. 4A). A similar pattern was observed in hFF, where *NANOG* expression increased by day 7 of culture in OM and significantly decreased by day 21 (Fig. 4C). hGFs displayed a different response, as they did not upregulate *NANOG* expression by day 7. However, in these cells we detected an extremely high *NANOG* expression at day 14, followed by its abrupt downregulation by day 21 (Fig. 4B). Less dramatic fluctuations were detected in the expression of *NANOG* in all cell types cultured in adipogenic conditions (Fig. 4E–G). hDPSCs and hFFs displayed an upregulation of *NANOG* expression by day 7, followed by its downregulation by day 21 (Fig. 4E,G), similarly to what was observed upon incubation with OM. Of notice, the upregulation of *NANOG* in hFFs in AM was much less pronounced than that observed in OM (Fig. 4C,G). hGF cultured in AM displayed a modest modulation of *NANOG* expression, characterized by a mild, upregulation at day 7 and downregulation at day 14. The observed difference in the levels of *NANOG* expression in control conditions at day 0, with hFFs expressing lower levels (approx. 50% less) of NANOG compared to hDPSCs and hGFs, do not obviously correlate with the modulation of the expression of this gene throughout osteogenic and adipogenic differentiation (Fig. 4D,H).

**Figure 5:**
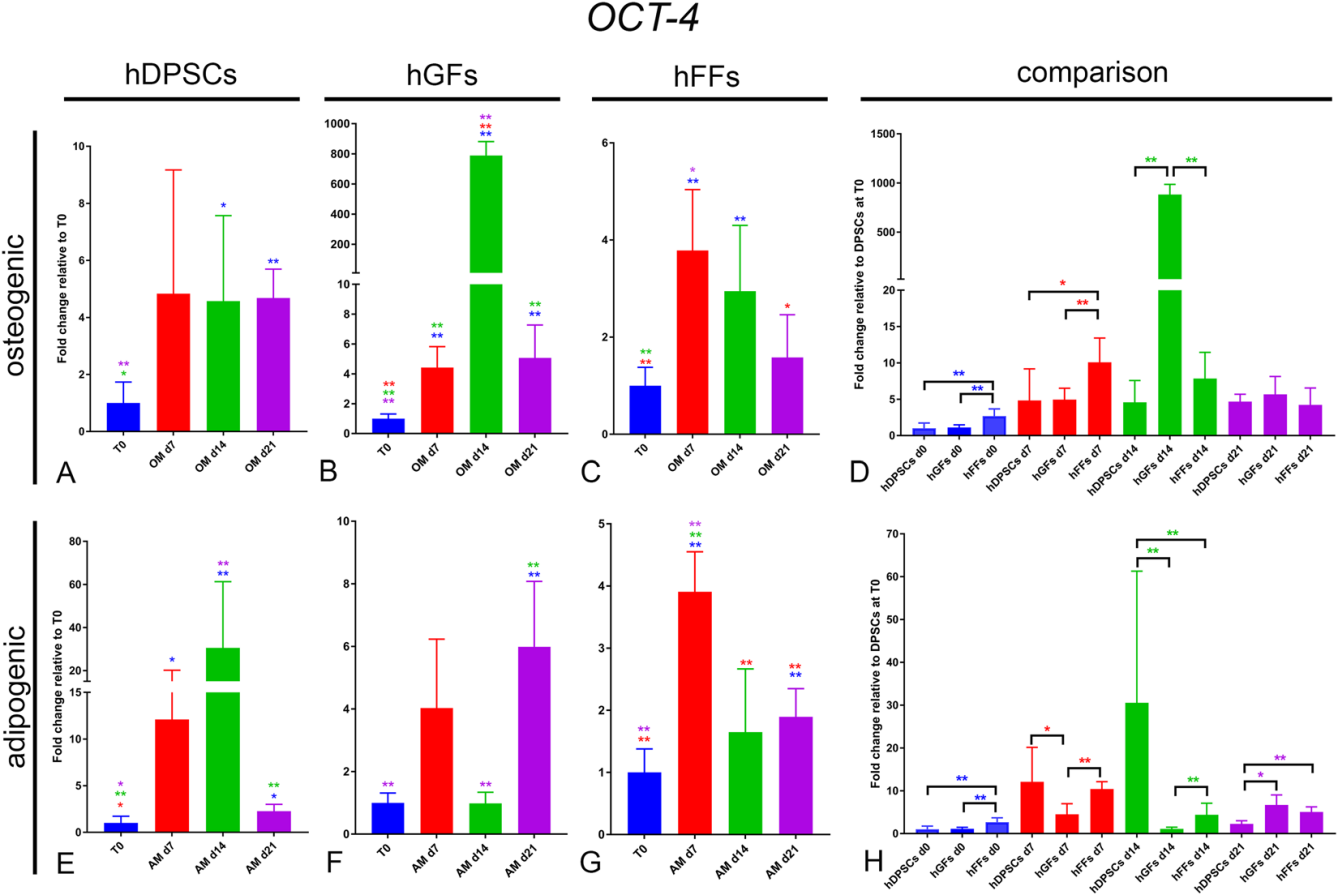
Gene expression levels of *OCT*-4 in cultured hDPSCs (**A**), hGFs (**B**) and hFFs (**C**) for 0, 7, 14 and 21 days in presence of OM and AM, relative to the respective expression levels at T0. (**D**) Expression levels of *OCT*-4 normalized for *OCT*-4 expression in hDPSCs at T0. Asterisks: Mann Whitney – U/Wilcox Rank Sum Test, *p < 0.05; **p < 0.01. Colour of asterisks onto each column indicates the column used for the comparison. Abbreviations: hDPSCs, human dental pulp stem cells; hFF, human foreskin fibroblasts; hGF, human gingival fibroblasts; OM, osteogenic medium; AM, adipogenic medium.

OCT-4: *OCT4* expression displayed trends grossly similar to those observed for *NANOG* expression. In hDPSCs, an increase, although variable, in *OCT4* expression at day 7 in OM was followed, at the subsequent timepoints, by its generalized maintenance at levels 4 times higher than in control conditions (Fig. 5A). Interestingly, culture in AM led to a strong upregulation of *OCT4* expression by day 14, followed by its significant downregulation by day 21 (Fig. 5E). Expression of *OCT4* in hFFs showed a trend similar to that observed analysing *NANOG* expression (Fig. 5C). In this case, however, the modulation of *OCT4* expression between the different time points in hFFs was less pronounced (Fig. 5C). Similar to *NANOG*, *OCT4* expression in hGFs cultured in OM displayed a great increase at day 14, followed by a sudden downregulation at day 21 (Fig. 5B). hGF cultured in AM displayed an opposite but much more modest modulation of *OCT4* expression (Fig. 5F). In these cells, *OCT4* was mildly upregulated at day 7, downregulated at day 14 and upregulated again at day 21 (Fig. 4B). In contrast to what observed for *NANOG*, at the basal level, hFFs displayed a 2-fold higher expression of *OCT4*, compared to hDPSCs and hGFs (Fig. 5D,H).

#### Gene expression analysis of osteogenic and odontogenic markers

Runt Related Transcription Factor 2 (*RUNX2*), alkaline phosphatase (*ALP*) and osterix (*SP7/OSX*) are well-established osteogenic markers^28–30^. RUNX2 is crucial for the formation of mineralized tissues, and is generally used as a marker of early phases of osteogenic differentiation. *ALP* is a widely expressed hydrolase enzyme and plays a key role in the mineralization of hard tissues, as the hydrolysis of phosphate esters supplies free phosphate that is required for the creation of hydroxyapatite crystals^31,32^. OSX is encoded by the *SP7* gene and is an osteoblast-specific transcription factor^29,33^. *DSPP* codes for dentin sialophosphoprotein, a key component of the dentin matrix, expressed at highest levels by odontoblasts^34^.

**Figure 6:**
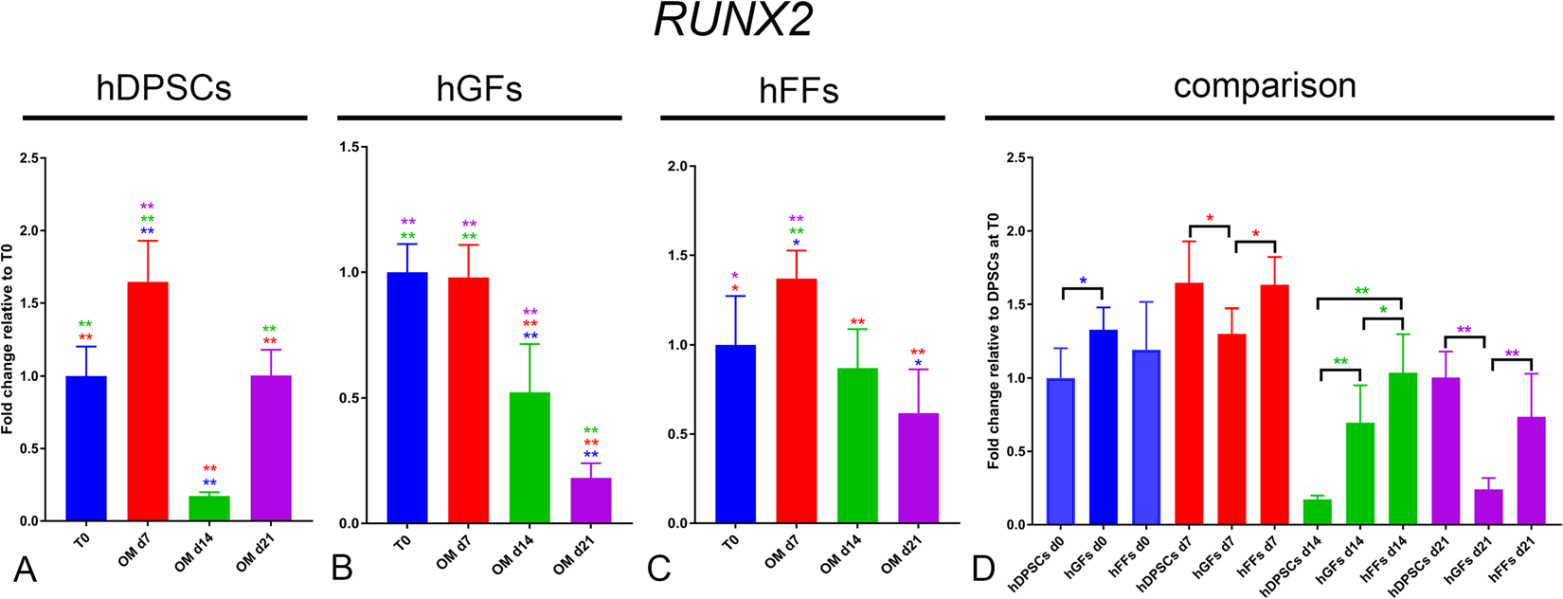
Gene expression levels of *RUNX2* in cultured hDPSCs (**A**), hGFs (**B**) and hFFs (**C**) for 0, 7, 14 and 21 days in presence of OM, relative to the respective expression levels at T0. (**D**) Expression levels of *RUNX2* normalized for *RUNX2* expression in hDPSCs at T0. Asterisks: Mann Whitney – U/Wilcox Rank Sum Test, *p < 0.05; **p < 0.01. Colour of asterisks onto each column indicates the column used for the comparison. Abbreviations: hDPSCs, human dental pulp stem cells; hFF, human foreskin fibroblasts; hGF, human gingival fibroblasts; OM, osteogenic medium.

RUNX2: hDPSCs and hFFs incubated with OM exhibited upregulation of *RUNX*2 expression levels at day 7. In both groups *RUNX*2 expression decreased at day 14, and increased subsequently in DPSCs at day 21 (Fig. 6A,C,D). In hGFs, incubation in OM did not lead to any increase in *RUNX2* expression, and ultimately led to its significant downregulation at day 14 and 21 (Fig. 6B). At basal levels the expression of *RUNX2* was comparable in all groups, while it was surprisingly lower in hDPSCs at day 14 (Fig. 6D). At day 21, RUNX2 expression was significantly higher in hDPSCs and hFFs, compared to hGFs (Fig. 6D).

**Figure 7:**
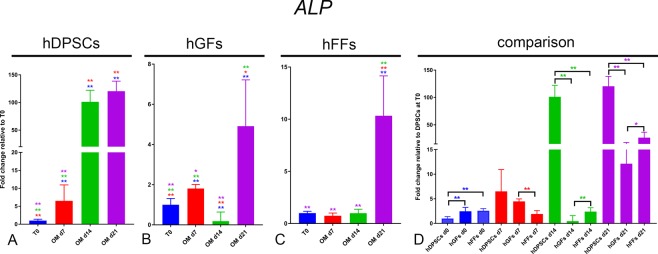
Gene expression levels of *ALP* in cultured hDPSCs (**A**), hGFs (**B**) and hFFs (**C**) for 0, 7, 14 and 21 days in presence of OM, relative to the respective expression levels at T0. (**D**) Expression levels of *ALP* normalized for *ALP* expression in hDPSCs at T0. Asterisks: Mann Whitney – U/Wilcox Rank Sum Test, *p < 0.05; **p < 0.01. Colour of asterisks onto each column indicates the column used for the comparison. Abbreviations: hDPSCs, human dental pulp stem cells; hFF, human foreskin fibroblasts; hGF, human gingival fibroblasts; OM, osteogenic medium.

ALP: An increase in *ALP* expression was observed in all cell groups cultured in presence of OM (Fig. 7). In hDPSCs *ALP* upregulation was already significant at day7, and reached a >100-fold increase by day 14, maintained at day 21 (Fig. 7A). The increase was much more modest in hGFs and hFFs, where *ALP* expression reached a maximum of a 5-fold and 10-fold increase compared to time 0, respectively (Fig. 7B,C). Basal levels of *ALP* were comparable among all groups, although hGFs and hFFs displayed a mildly higher expression when compared to hDPSCs. From day 14, *ALP* levels were significantly higher in hDPSCs than in hFFs and hGFs (Fig. 7D).

**Figure 8:**
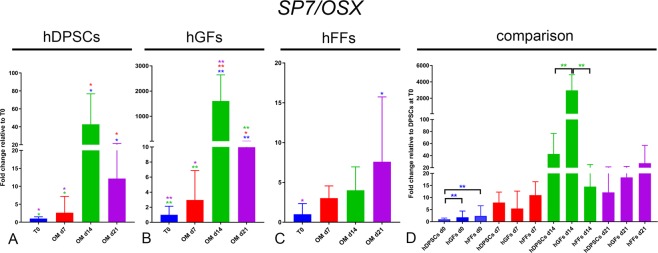
Gene expression levels of *OSX* in cultured hDPSCs (**A**), hGFs (**B**) and hFFs (**C**) for 0, 7, 14 and 21 days in presence of OM, relative to the respective expression levels at T0. (**D**) Expression levels of *OSX* normalized for *OSX* expression in hDPSCs at T0. Asterisks: Mann Whitney – U/Wilcox Rank Sum Test, *p < 0.05; **p < 0.01. Colour of asterisks onto each column indicates the column used for the comparison. Abbreviations: hDPSCs, human dental pulp stem cells; hFF, human foreskin fibroblasts; hGF, human gingival fibroblasts; OM, osteogenic medium.

SP7/OSX: Incubation with OM induced a significant upregulation of *SP7* expression already at day 7 in hDPSCs and hGFs (Fig. 8A,B). At day 14, hDPSCs and hGFs showed a massive *SP7* upregulation (Fig. 8A,B), while the levels detected in hFFs remained constant (Fig. 8C). At day 21, hDPSCs and hGFs showed a downregulation of *SP7* expression, which however remained significantly higher than that observed at T0 (Fig. 8A–C). At basal levels, hDPSCs displayed a slightly lower expression of *SP7* (Fig. 8D).

**Figure 9:**
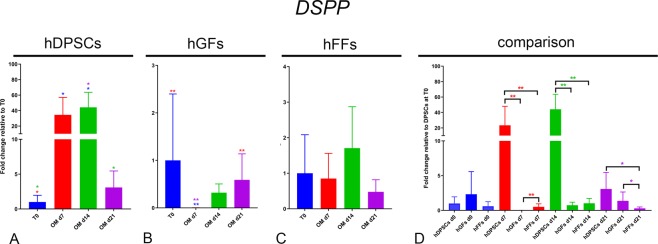
Gene expression levels of *DSPP* in cultured hDPSCs (**A**), hGFs (**B**) and hFFs (**C**) for 0, 7, 14 and 21 days in presence of OM, relative to the respective expression levels at T0. (D) Expression levels of *DSPP* normalized for *OSX* expression in hDPSCs at T0. Asterisks: Mann Whitney – U/Wilcox Rank Sum Test, *p < 0.05; **p < 0.01. Colour of asterisks onto each column indicates the column used for the comparison. Abbreviations: hDPSCs, human dental pulp stem cells; hFF, human foreskin fibroblasts; hGF, human gingival fibroblasts; OM, osteogenic medium.

DSPP: *DSPP* expression was significantly and progressively upregulated in hDPSCs at 7 and 14 days of culture in osteogenic conditions, and it was then downregulated to basal levels by day 21 (Fig. 9A). No significant increase in *DSPP* expression could be detected in hGFs and hFFs (Fig. 9B,C). On the contrary, *DSPP* expression became transiently undetectable in hGFs upon incubation in OM (Fig. 9B). At all stages from day 7 to day 21, *DSPP* levels were significantly higher in hDPSCs compared to both hGFs and hFFs (Fig. 9D).

#### Gene expression analysis of adipogenic markers

Peroxisome proliferator-activated receptor γ2 (*PPAR-γ2*) and lipoprotein lipase (*LPL*) are used as adipogenic differentiation marker genes. PPAR-γ2 is considered as a master regulator of adipogenesis^28^, while LPL is involved in lipid transport and provides glycerol and free fatty acids by catalysing the hydrolysis of triglycerides^35^.

**Figure 10:**
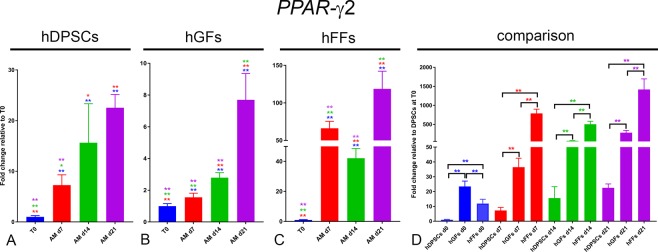
Gene expression levels of *PPAR-γ2* in cultured hDPSCs (**A**), hGFs (**B**) and hFFs (**C**) for 1, 7, 14 and 21 days in presence of AM, relative to the respective expression levels at T0. (**D**) Expression levels of *PPAR-γ2* normalized for *PPAR-γ2* expression in hDPSCs at T0. Asterisks: Mann Whitney – U/Wilcox Rank Sum Test, *p < 0.05; **p < 0.01. Colour of asterisks onto each column indicates the column used for the comparison. Abbreviations: hDPSCs, human dental pulp stem cells; hFF, human foreskin fibroblasts; hGF, human gingival fibroblasts; AM, adipogenic medium.

PPAR-γ2: *PPAR-γ2* expression was upregulated in all groups cultured in the presence of AM (Fig. 10). This upregulation was extremely pronounced in hFFs, where *PPAR-γ2* expression reached levels 70-fold higher than at T0 already after 7 days, and remained high throughout the differentiation period, with a decrease at day 14 (Fig. 10C). *PPAR-γ2* was also upregulated in hDPSCs and hGFs cultured in AM, albeit to a lower extent. In these two groups, *PPAR-γ2* expression increased progressively from day 0 to day 21 (Fig. 10A,B). Interestingly, expression of *PPAR-γ2* was significantly higher in fibroblasts compared to hDPSCs already at time 0, with *PPAR-γ2* expression in hGFs even >20-fold higher than that detected in hDPSCs (Fig. 10D).

**Figure 11:**
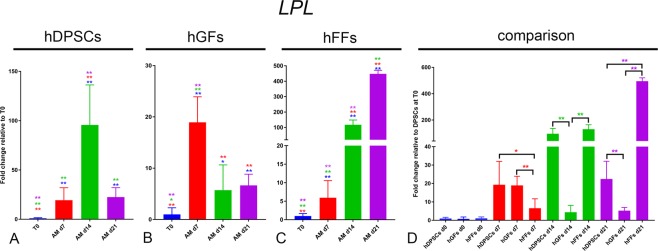
Gene expression levels of *LPL* in cultured hDPSCs (**A**), hGFs (**B**) and hFFs (**C**) for 0, 7, 14 and 21 days in presence of AM, relative to the respective expression levels at T0. (**D**) Expression levels of *LPL* normalized for *LPL* expression in hDPSCs at T0. Asterisks: Mann Whitney – U/Wilcox Rank Sum Test, *p < 0.05; **p < 0.01. Colour of asterisks onto each column indicates the column used for the comparison. Abbreviations: hDPSCs, human dental pulp stem cells; hFF, human foreskin fibroblasts; hGF, human gingival fibroblasts; AM, adipogenic medium.

LPL: *LPL* expression was upregulated in all three groups cultured in AM (Fig. 11). In hDPSCs *LPL* expression peaked at day 14, to then decrease at day 21 (Fig. 11A). In hGFs the upregulation observed at day 7 was followed by a progressive downregulation at day 14 and day 21 (Fig. 11B). hFFs showed an opposite trend, as they displayed a continuos upregulation of *LPL* expression from day 0 to day 21, with a peak of 400-fold increase at the latest timepoint (Fig. 11C). In contrast to what was observed with *PPAR-γ2*, the expression levels of *LPL* before any treatment were comparable among the three groups (Fig. 11D).

## Discussion

Carious and periodontal diseases such as periodontitis, fractures, genetic defects or aging can lead to tooth damage and loss, thus decreasing the quality of life^36^. So far in dental clinical practice the treatment of choice for replacing missing teeth are osseointegrated dental implants^37^, while the treatment of a carious lesion consists of the removal of the infected hard tissue part and its replacement by composite resins^38^. When the bacterial infection reaches the dental pulp, root canal therapy is the treatment of choice, in which the pulp is substituted by synthetic filling materials^39^. Regenerative dentistry represents an alternative solution for the repair of dental tissues using a variety of techniques and therapeutic approaches^40^. For example, methods used nowadays include injection of stem cells and/or soluble molecules such as growth factors^41^. Therefore, the goal of regenerative dentistry is to achieve partial or complete regeneration of the damaged or missing dental tissues, thus restoring their biological function and structure^40^. Various promising *in vivo* studies have already demonstrated the potential of human dental pulp stem cells (hDPSCs) for regenerative purposes, such as alveolar and mandibular bone regeneration in patients or the reestablishment of dental pulp and mineralized tissues in dogs^42,43^. However, there is a need for alternatives since teeth do not always constitute an ideal cell source: the supply of hDPSCs is limited and bound to the extraction of the respective, healthy tooth^8^. Fibroblasts from different organs can be more accessible, as they can be obtained from a plethora of surgical procedures, and have been shown to share many similarities with mesenchymal stem cells (MSCs) such as the multilineage differentiation potential^19^. Fibroblasts for various sources were already analysed for their regenerative potential. Human gingival fibroblasts (hGFs) are easily obtainable from the gingiva that is often resected during general dental treatments^44^. We have recently shown that both hGFs and hDPSCs are able to attract vessels when seeded into silk fibroin scaffolds, and therefore may improve healing and regeneration of damaged tissues^45^. While there is still little information on the stem cell properties and differentiation potential of hGFs^46^, several *in vitro* studies have shown that these cells are able to form osteogenic, chondrogenic and adipogenic tissues^47,48^. Human foreskin fibroblasts (hFFs) are accessible after circumcision/biopsies and are also capable to differentiate into bone, cartilage and fat^49^. Several studies have indicated that both gingival and foreskin tissues contain subpopulations of mesenchymal progenitor/stem cells that allow cytodifferentiation into multiple lineages^50–52^. However, it is not clear if distinct cell types exist within these two tissues, since hGFs and hFFs are barely distinguishable from MSCs regarding phenotype, expression of specific markers and immunosuppression responses^53,54^.

Cell differentiation follows a shift in gene expression, a process involving the coordinated action of transcription factors, non-coding microRNA, DNA methylation, histone modifications and other chromatin remodeling activities^55,56^. *NANOG* and *OCT-4* are both well-established embryonic stem cell (ESC) markers. They play a major role in the maintenance of the pluripotent state of ESCs and are down-regulated as the cells become more committed^22,27^. Although previous studies suggested that these genes were no longer expressed in adult stem cells^23^, more recent findings showed that their expression persists in MSCs^24^. We detected low levels of *NANOG* and *OCT4* in basal conditions in hDPSCs, hGFs and hFF, followed by a significant initial upregulation and successive modulation of their expression mostly during osteogenic differentiation in all three cell populations. Importantly, the dynamic of the modulation of these genes in the three cell types was very diverse, showing a cell-type-specific regulation of stemness-related genes upon differentiation, thus indicating that the expression of *NANOG* and *OCT-4* is not simply increased or decreased in equal measure as differentiation occurs or subsides. These results apparently contradict previous findings showing down-regulation of this gene during cytodifferentiation^23,57^. However, recent studies suggested that increased *OCT-4* expression enhances the ability of MSCs to differentiate into osteogenic and adipogenic lineages^58,59^, possibly priming loci coding for factors fundamental for lineage commitment^60^. MSCs have been shown to differ in the expression of ESC markers depending on the source from which they were obtained^58^. It is likely that the decision if a cell will differentiate or remain quiescent and self-renew is the result of the interplay with many other transcription factors and pathways and a question of increased and decreased expression of genes over the course of time rather than a simple switch on or off^22,61^. In this regard, all groups modulated the expression of *OCT4* and *NANOG* both in osteogenic and adipogenic conditions. hGFs displayed a striking synchronized increase in the expression of these two genes after two weeks of incubation in osteogenic medium, a dynamic completely different from that of hDPSCs and hFF, which might be correlated with the observed differences in differentiation potential. In fact, hDPSCs and hFFs, but not hGFs, were able to form mineralization nodules upon osteogenic induction *in vitro*. This correlates with the different modulation of osteogenic markers observed in hDPSCs and hFF, and hGFs. *RUNX2* is known as a master control gene in osteoblastic differentiation as it plays a crucial role in the differentiation of MSCs into preosteoblasts^62^. *SP7/OSX* is involved in the maturation of preosteoblasts into mature osteoblasts^29^. ALP plays an important role in the mineralization process^31^ and is often used as a marker for osteogenic differentiation. In osteogenic conditions, both hDPSCs and hFF showed sustained *RUNX2* expression, a moderate peak of *SP7/OSX* expression at day 14, and progressive upregulation of *ALP*. hGFs failed to maintain *RUNX2* expression and to upregulate *ALP*. However, hGFs showed a striking peak in *SP7/OSX* expression at day14, associated with a major upregulation of *OCT4* and *NANOG*. Although previous *in vitro* studies have demonstrated that hGFs are capable to form mineralized deposits in osteogenic media concentrations that differ from those used for the present study^46^, the observed expression patterns might be indicative of a cell-specific incapability to pursue the full osteogenic differentiation path. In this regard, it has been shown that *SP7/OSX* overexpression can induce osteogenic differentiation in murine embryonic stem cells and murine bone marrow stromal cells, but not in fibroblasts^63^. Importantly, hDPSCs were the only cell type upregulating DSPP expression when cultured in osteogenic conditions. This is in accordance with their tissue of origin and their known ability to give rise to odontoblasts^64,65^. The observed dynamic modulation of *RUNX2*, *OSX* and *ALP* also correlates with an odontoblastic differentiation program. During murine tooth development, *RUNX2* and *OSX* are highly expressed in immature odontoblasts, while *RUNX2* is downregulated upon terminal differentiation^66^. *ALP*, on the contrary, is expressed at high levels also in mature odontoblasts^67^. These observations suggest that hDPSCs cultured in osteogenic conditions actually show an odontoblastic-like differentiation dynamic. The expression of *PPAR-γ2*, a transcription factor essential in the formation of adipocytes^28^, and *LPL*, which is expressed in preadipocytes and plays a crucial role in lipid metabolism and concentration of triglycerides^35^, was analyzed in order to assess the adipogenic differentiation potential of the three cell populations. In adipogenic culture conditions, significant upregulation of *PPAR-γ2* and *LPL* expression was observed in all experimental groups already at early time points. This upregulation was particularly pronounced in hFFs. Histological staining with Oil Red O revealed lipid droplets in hFFs and hGFs, but not in hDPSCs. Similarly, only hFFs and hGFs showed a shift from fibroblastic/spindle to spherical cell shape, which represents a clear sign of adipogenic differentiation^28,35,68^. *LPL* expression was significantly higher in hFFs, but not in hGF, when compared to hDPSCs, despite the clearly higher adipogenic potential of both these fibroblastic populations (as indicated by Oil Red O stainings). Fibroblasts expressed significantly higher levels of *PPAR-γ2* already in basal conditions, with hGFs expressing over 20-fold more *PPAR-γ2* than hDPSCs. This higher expression was then maintained throughout the differentiation period. These results thus indicate that hFFs and hGFs possess a significantly higher adipogenic potential compared to hDPSCs. Nevertheless, *in vitro* differentiation assays do not constitute a physiological environment and therefore it is not clear whether the observed changes during cytodifferentiation are rather caused by a temporary up-regulation of tissue-specific genes due to artificial *in vitro* concentrations of substances and if they can truly be translated to an *in vivo* situation^18^. In conclusion, the present findings support the idea of using fibroblasts for regenerative purposes based on their multilineage differentiation potential. Both hGFs and hFFs contain multipotent progenitors that are able to form osteogenic and adipogenic tissues and are more prone towards adipogenic differentiation when compared to hDPSCs. However, hDPSCs might represent a more appropriate cell population for regenerative purposes involving bone and dental tissues.

## Materials and Methods

### Collection of human cells

The procedure for anonymized human dental pulp stem cells (hDPSCs) and human gingival fibroblasts (hGFs) collection at the Zentrum für Zahnmedizin, Zürich, was approved by the Kantonale Ethikkommission of Zurich (reference number 2012–0588) and the patients gave their written informed consent. All procedures were performed according to the current guidelines. All surgical procedures and tooth extractions were performed by professional surgeons and dentists. Human foreskin fibroblasts (hFFs) were purchased from ATCC (ATCC, Manassas VA, USA). Human dental pulp stem cells (hDPSCs) were isolated from the dental pulp of extracted wisdom teeth of healthy patients as previously described^45^. The dental pulps were enzymatically digested for one hour at 37 °C in a solution of collagenase (3 mg/mL; Life Technologies Europe BV, Zug ZG, Switzerland) and dispase (4 mg/mL; Sigma-Aldrich Chemie GmbH, Buchs SG, Switzerland). A filtered single-cell suspension was plated in a 40 mm Petri dish with hDPSC growth medium containing DMEM/F12 (Sigma-Aldrich Chemie GmbH, Buchs SG, Switzerland) with 10% fetal bovine serum (FBS) (PAN Biotech GmbH, Aidenbach, Germany), 1% penicillin/streptomycin (P/S) (Sigma-Aldrich Chemie GmbH, Buchs SG, Switzerland), 1% L-glutamine (Sigma-Aldrich Chemie GmbH, Buchs SG, Switzerland), and 0.5 μg/ml fungizone (Life Technologies Europe BV, Zug ZG, Switzerland) after washing away the enzyme solution. Cells were passaged at 80–90% confluence and expanded in the same growth medium. Gingival fibroblasts (hGFs) were isolated from healthy parts of gingiva collected from biopsies, as previously described^45^. Gingival tissues were washed in phosphate buffered saline (PBS) (Life Technologies Europe B.V., Zug ZG, Switzerland), sectioned into small pieces and placed in 35 mm Petri dishes (TPP Techno Plastic Products AG, Trasadingen SH, Switzerland) for the outgrowth of human gingival fibroblasts (hGFs). The fibroblast growth medium was composed by high glucose DMEM/F12 (Life Technologies Europe B.V., Zug ZG, Switzerland), 10% FBS, 1% P/S, and 1% HEPES (Sigma-Aldrich Chemie GmbH, Buchs SG, Switzerland). hGFs were expanded in DMEM high glucose (Thermo Fisher Scientific AG, Reinach BL, Switzerland) supplemented with 10% FBS, 1% P/S, and 1% HEPES (Thermo Fisher Scientific AG, Reinach BL, Switzerland). Cells were passaged at 80–90% confluence.

### Cell culture and differentiation

Cells were expanded as monolayers on T-150 culture flasks (Sarsted AG, Switzerland) in Dulbecco’s Modified Eagle Medium: Nutrient Mixture F-12 (DMEM/F12; ThermoFisher/Life Technologies, Switzerland) supplemented with 10% Foetal Bovine Serum (FBS, Bioswisstech AG, Switzerland), 100 U/ml penicillin/streptomycin (Sigma-Aldrich/Merck, Darmstadt, Germany), and Amphotericin B 0.25 μg/μL (ThermoFisher Scientific, Switzerland) incubated at 37 °C in 5% CO_2._ The medium was replaced every second day. Cells were passaged once a confluence of 70–80% was reached. Cells were washed once with phosphate buffered saline (PBS) before trypsin was added for 3 min at 37 °C for their detachment. Trypsin was blocked by addition of 5 volumes of DMEM/F12 supplemented with 10% FBS. The cells were then centrifuged and seeded into T25 flasks (Sarsted AG, Switzerland) for the differentiation assays. 20’000 cells per well were seeded onto 24-well-plates for histological staining, while for gene expression analysis, 250’000 cells were seeded onto T25 plates.

The osteogenic differentiation medium consisted of DMEM supplemented with Ascorbic Acid (200 μM), β-Glycerolphosphate (10 mM), Dexamethasone (10 nM) (Sigma-Aldrich/Merck, Darmstadt, Germany), and Amphotericin B 0.25 μg/μL (ThermoFisher Scientific, Switzerland). The adipogenic differentiation medium consisted of DMEM (1 ml) supplemented with Dexamethasone (1 μM), IBMX (0.5 mM), Indomethacin (200 μM), Insulin (10 μM) (Sigma-Aldrich/Merck, Darmstadt, Germany) and Amphotericin B 0.25 μg/μL. Cells were cultured for 21 days in osteogenic medium (OM) and adipogenic medium (AM). Cells were collected from the T25 flasks on day 0 (plating day), 7, 14 and 21 and used for RNA extraction. Cells cultured on 24-wells plates were cultured for 21 days, stained (see following paragraph) and examined under a bright-field microscope.

### Stainings

*Alizarin Red S* staining was performed to identify extracellular calcium deposits of cells differentiated into osteoblasts. Alizarin Red S powder was dissolved in distilled water, pH 4.2. Cells were washed with PBS, fixed with 4% PFA for 30 min, washed with distilled water and finally Alizarin Red S staining solution was added to each well for 45 min at room temperature in the dark. Thereafter wells were washed with deionized water and then PBS was added. The cells were viewed under a bright-field microscope, where calcium deposits exhibited a bright orange-red color. *Oil Red O* staining was performed to identify lipids in cells differentiated into adipocytes. 300 mg of Oil Red O powder were added to 100 ml of 99% isopropanol and then mixed with deionized water and filtered through a funnel. Cells were washed with PBS, fixed with 4% PFA for 30 min, washed again with deionized water, 60% isopropanol was added for 2–5 min and after isopropanol aspiration Oil Red O was added for 5 min. Thereafter, the wells were rinsed with tap water, hematoxylin counterstain was performed for 1 min and cells were rinsed with warm tap water. The cells were viewed under a bright-field microscope, where lipids exhibited a red color while the nuclei of cells were blue.

Stainings were quantified by measuring the proportion of Alizarin-Red and Oil-Red-O positive area over the total area imaged, using Fiji^69^. 3 independent samples were analysed for each cell type.

### Gene expression analysis

#### Collection of cells and snap-freezing

Cells were collected by trypsinization at day 0, 7, 14 and 21, snap-frozen in liquid nitrogen and stored at −80 °C.

#### RNA isolation and purification

The RNA isolation on snap-frozen cells from the differentiation assays using the RNeasy Plus Universal Mini Kit was performed according to the instructions given (Qiagen AG, Hombrechtikon ZH, Switzerland).

#### cDNA synthesis

Reverse transcription of the isolated RNA was performed using the iScript™ cDNA synthesis Kit and according to the instructions given (Bio-Rad Laboratories AG, Cressier FR, Switzerland). Briefly, 1000 ng of RNA were used for reverse transcription into cDNA. Nuclease-free water was added to add up to a total of 15 μl. 4 μl of 5x iScript reaction mix and 1 μl of iScript reverse transcriptase were added per sample in order to obtain a total volume of 20 μl. The reaction mix was then incubated for 5 min at 25 °C, for 30 min at 42 °C and for 5 min at 85 °C using a Biometra TPersonal Thermocycler (Biometra AG, Göttingen, Germany).

#### Quantitative real-time PCR

The 3-step quantitative real-time PCRs were performed using an Eco Real-Time PCR System (Illumina Inc., San Diego CA, USA). Expression level analysis of *GAPDH* (housekeeping gene), *ALP*, *SP7*, *RUNX2*, *PPAR-γ2*, *LPL*, *OCT-4* and *NANOG* were carried out using the SYBR® Green PCR Master Mix (Applied Biosystems, Carlsbad CA, USA) in combination with oligonucleotide primers (Table 1). Using MicroAmp® Fast Optical 48-Well Reaction Plates (Applied Biosystems, Carlsbad CA, USA), 5 μl of SYBR® Green PCR Master Mix reverse and forward primers (200 nM), and 2 ng of template cDNA were added to each well. The thermocycling conditions were: 95 °C for 10 min, followed by 40 cycles of 95 °C for 15 sec, 55 °C for 30 sec and 60 °C for 1 min. Melt curve analysis was performed at 95 °C for 15 sec, 55 °C for 15 sec and 95 °C for 15 sec. Expression levels were calculated by the comparative ΔΔCt method (2^−ΔΔCt^ formula), after being normalized to the Ct-value of the *GAPDH* housekeeping gene. Gene expression analysis was performed on 6 independent samples per condition. Samples were always compared one-vs-one using the Mann Whitney - U/Wilcox Rank Sum Test (Graph Pad Prism 8.0).

**Table 1:** List of the oligonucleotide primers used.

Gene	Accession no.	Forward primer 5′-3′	Reverse primer 5′-3′	Amplicon length (bps)
*GAPDH*	NM_002046.5	AGGGCTGCTTTTAACTCTGGT	CCCCACTTGATTTTGGAGGGA	205
*NANOG*	NM_024865.3	TTTGTGGGCCTGAAGAAAACT	AGGGCTGTCCTGAATAAGCAG	115
*OCT-4*	NM_002701.5	CTTTCTCAGGGGGACCAGTG	GGGACCGAGGAGTACAGTGC	102
*RUNX2*	NG_008020.1	GCCAGGGTCTAGGAGTTGTT	ACCCACCACCCTATTTCCTG	212
*ALP*	NM_000478.5	ATGAAGGAAAAGCCAAGCAG	ATGGAGACATTCTCTCGTTC	276
*SP7 (OSX)*	NM_152860.1	CCTCTGCGGGACTCAACAAC	AGCCCATTAGTGCTTGTAAAGG	127
*DSPP*	NM_014208.3	TTTGGGCAGTAGCATGGG	CCATCTTGGGTATTCTCT	181
*PPAR-γ2*	NM_138712.3	GAACGACCAAGTAACTCTCC	CGCAGGCTCTTTAGAAACTCC	137
*LPL*	NM_000237.2	ACGGCATGTGAATTCTGTGA	GGATGTGCTATTTGGCCACT	200

Abbreviations: GAPDH, Glyceraldehyde-3-phosphate dehydrogenase; *OCT*-4, Octamer-binding transcription factor 4; RUNX2, Runt-related transcription factor 2; ALP, Alkaline phosphatase; SP7(OSX), Sp7 Transcription factor; DSPP, Dentin Sialophosphoprotein; PPAR-γ2, Peroxisome proliferator-activated receptor; LPL, Lipoprotein lipase.

### Ethical approval and informed consent

The procedure for anonymized human dental pulp stem cells (hDPSCs) and human gingival fibroblasts (hGFs) collection at the Zentrum für Zahnmedizin, Zürich, was approved by the Kantonale Ethikkommission of Zurich (reference number 2012–0588; confirmed 2017–00932) and the patients gave their written informed consent. All procedures were performed according to the current guidelines. All surgical procedures and tooth extractions were performed by professional surgeons and dentists. Human foreskin fibroblasts (hFFs) were purchased from ATCC (ATCC, Manassas VA, USA).

## Data Availability

The datasets generated during and/or analysed during the current study are available from the corresponding author on reasonable request.

## References

[1] Signer RAJ, Morrison SJ (2013). Mechanisms that regulate stem cell aging and life span. Cell Stem Cell.

[2] Mitsiadis TA, Barrandon O, Rochat A, Barrandon Y, De Bari C (2007). Stem cell niches in mammals. Exp. Cell Res..

[3] Barry FP, Murphy JM (2004). Mesenchymal stem cells: clinical applications and biological characterization. Int J Biochem Cell Biol.

[4] Heino TJ, Hentunen TA (2008). Differentiation of osteoblasts and osteocytes from mesenchymal stem cells. Curr Stem Cell Res Ther.

[5] Beyer Nardi, N. & da Silva Meirelles, L. Mesenchymal stem cells: isolation, *in vitro* expansion and characterization. H*andb Exp Pharmacol* 249–282 (2006). 16370331

[6] Dominici M (2006). Minimal criteria for defining multipotent mesenchymal stromal cells. The International Society for Cellular Therapy position statement. Cytotherapy.

[7] Russell KC (2010). *In vitro* high-capacity assay to quantify the clonal heterogeneity in trilineage potential of mesenchymal stem cells reveals a complex hierarchy of lineage commitment. Stem Cells.

[8] Gronthos S, Mankani M, Brahim J, Robey PG, Shi S (2000). Postnatal human dental pulp stem cells (DPSCs) *in vitro* and *in vivo*. Proc. Natl. Acad. Sci. USA.

[9] Huang GT-J, Gronthos S, Shi S (2009). Mesenchymal stem cells derived from dental tissues vs. those from other sources: their biology and role in regenerative medicine. J. Dent. Res..

[10] Tatullo M, Marrelli M, Paduano F (2015). The regenerative medicine in oral and maxillofacial surgery: the most important innovations in the clinical application of mesenchymal stem cells. Int J Med Sci.

[11] Mitsiadis TA, Orsini G, Jimenez-Rojo L (2015). Stem cell-based approaches in dentistry. Eur Cell Mater.

[12] Pierdomenico L (2005). Multipotent mesenchymal stem cells with immunosuppressive activity can be easily isolated from dental pulp. Transplantation.

[13] Zhang W, Walboomers XF, Shi S, Fan M, Jansen JA (2006). Multilineage differentiation potential of stem cells derived from human dental pulp after cryopreservation. Tissue Eng..

[14] Mitsiadis TA, Feki A, Papaccio G, Catón J (2011). Dental pulp stem cells, niches, and notch signaling in tooth injury. Adv. Dent. Res..

[15] Huang GT-J (2010). Stem/progenitor cell-mediated de novo regeneration of dental pulp with newly deposited continuous layer of dentin in an *in vivo* model. Tissue Eng Part A.

[16] Davies OG, Cooper PR, Shelton RM, Smith AJ, Scheven BA (2015). A comparison of the *in vitro* mineralisation and dentinogenic potential of mesenchymal stem cells derived from adipose tissue, bone marrow and dental pulp. J. Bone Miner. Metab..

[17] Haniffa MA, Collin MP, Buckley CD, Dazzi F (2009). Mesenchymal stem cells: the fibroblasts’ new clothes?. Haematologica.

[18] Hematti P (2012). Mesenchymal stromal cells and fibroblasts: a case of mistaken identity?. Cytotherapy.

[19] Denu RA (2016). Fibroblasts and Mesenchymal Stromal/Stem Cells Are Phenotypically Indistinguishable. Acta Haematol..

[20] Lv F-J, Tuan RS, Cheung KMC, Leung VYL (2014). Concise review: the surface markers and identity of human mesenchymal stem cells. Stem Cells.

[21] Haniffa MA (2007). Adult human fibroblasts are potent immunoregulatory cells and functionally equivalent to mesenchymal stem cells. J. Immunol..

[22] Boiani M, Schöler HR (2005). Regulatory networks in embryo-derived pluripotent stem cells. Nat. Rev. Mol. Cell Biol..

[23] Hart AH, Hartley L, Ibrahim M, Robb L (2004). Identification, cloning and expression analysis of the pluripotency promoting Nanog genes in mouse and human. Dev. Dyn..

[24] Riekstina U (2009). Embryonic stem cell marker expression pattern in human mesenchymal stem cells derived from bone marrow, adipose tissue, heart and dermis. Stem Cell Rev.

[25] Kerkis I (2006). Isolation and characterization of a population of immature dental pulp stem cells expressing OCT-4 and other embryonic stem cell markers. Cells Tissues Organs (Print).

[26] La Noce M (2014). Dental pulp stem cells: state of the art and suggestions for a true translation of research into therapy. J Dent.

[27] Shi G, Jin Y (2010). Role of Oct4 in maintaining and regaining stem cell pluripotency. Stem Cell Res Ther.

[28] James, A. W. Review of Signaling Pathways Governing MSC Osteogenic and Adipogenic Differentiation. *Scientifica*10.1155/2013/684736 (2013). 10.1155/2013/684736 PMC3874981 24416618

[29] Zhang C (2010). Transcriptional regulation of bone formation by the osteoblast-specific transcription factor Osx. J Orthop Surg Res.

[30] Otto F (1997). Cbfa1, a candidate gene for cleidocranial dysplasia syndrome, is essential for osteoblast differentiation and bone development. Cell.

[31] Štefková, K., Procházková, J. & Pacherník, J. Alkaline Phosphatase in Stem Cells. S*tem Cells Int* 2**015** (2015). 10.1155/2015/628368 PMC4342173 25767512

[32] Kim YH, Yoon DS, Kim HO, Lee JW (2012). Characterization of different subpopulations from bone marrow-derived mesenchymal stromal cells by alkaline phosphatase expression. Stem Cells Dev..

[33] Peng Y (2013). Characterization of Osterix protein stability and physiological role in osteoblast differentiation. Plos One.

[34] Yamakoshi Y (2008). Dentin Sialophophoprotein (DSPP) and Dentin. J Oral Biosci.

[35] Li Y (2014). Lipoprotein lipase: from gene to atherosclerosis. Atherosclerosis.

[36] Mitsiadis TA, Woloszyk A, Jiménez-Rojo L (2012). Nanodentistry: combining nanostructured materials and stem cells for dental tissue regeneration. Nanomedicine (Lond).

[37] Mitsiadis TA, Papagerakis P (2011). Regenerated teeth: the future of tooth replacement?. Regen Med.

[38] Ferracane JL (2011). Resin composite—State of the art. Dental Materials.

[39] Ricketts D (2001). Management of the deep carious lesion and the vital pulp dentine complex. Br Dent J.

[40] Mason C, Dunnill P (2008). A brief definition of regenerative medicine. Regen Med.

[41] Abou Neel EA, Chrzanowski W, Salih VM, Kim H-W, Knowles JC (2014). Tissue engineering in dentistry. Journal of Dentistry.

[42] Iohara K (2013). A novel combinatorial therapy with pulp stem cells and granulocyte colony-stimulating factor for total pulp regeneration. Stem Cells Transl Med.

[43] d’Aquino R (2009). Human mandible bone defect repair by the grafting of dental pulp stem/progenitor cells and collagen sponge biocomplexes. Eur Cell Mater.

[44] Egusa H, Sonoyama W, Nishimura M, Atsuta I, Akiyama K (2012). Stem cells in dentistry – Part I: Stem cell sources. Journal of Prosthodontic Research.

[45] Woloszyk A, Buschmann J, Waschkies C, Stadlinger B, Mitsiadis TA (2016). Human Dental Pulp Stem Cells and Gingival Fibroblasts Seeded into Silk Fibroin Scaffolds Have the Same Ability in Attracting Vessels. Front Physiol.

[46] Mostafa NZ (2011). *In Vitro* Osteogenic Induction Of Human Gingival Fibroblasts For Bone Regeneration. Open Dent J.

[47] Mitrano TI (2010). Culture and characterization of mesenchymal stem cells from human gingival tissue. J. Periodontol..

[48] Jin SH (2015). Isolation and characterization of human mesenchymal stem cells from gingival connective tissue. J. Periodont. Res..

[49] Lorenz K (2008). Multilineage differentiation potential of human dermal skin-derived fibroblasts. Exp. Dermatol..

[50] Huang H-I (2010). Multilineage differentiation potential of fibroblast-like stromal cells derived from human skin. Tissue Eng Part A.

[51] Toma JG, McKenzie IA, Bagli D, Miller FD (2005). Isolation and characterization of multipotent skin-derived precursors from human skin. Stem Cells.

[52] Bartsch G (2005). Propagation, expansion, and multilineage differentiation of human somatic stem cells from dermal progenitors. Stem Cells Dev..

[53] Wetzig A (2013). Differential marker expression by cultures rich in mesenchymal stem cells. BMC Cell Biol..

[54] Wada N, Bartold PM, Gronthos S (2011). Human foreskin fibroblasts exert immunomodulatory properties by a different mechanism to bone marrow stromal/stem cells. Stem Cells Dev..

[55] Li B, Carey M, Workman JL (2007). The role of chromatin during transcription. Cell.

[56] Chen K, Rajewsky N (2007). The evolution of gene regulation by transcription factors and microRNAs. Nat. Rev. Genet..

[57] Pierantozzi E (2011). Pluripotency regulators in human mesenchymal stem cells: expression of NANOG but not of OCT-4 and SOX-2. Stem Cells Dev..

[58] Trivanović D (2015). Mesenchymal stem cells of different origin: Comparative evaluation of proliferative capacity, telomere length and pluripotency marker expression. Life Sci..

[59] Han S-M (2014). Enhanced proliferation and differentiation of Oct4- and Sox2-overexpressing human adipose tissue mesenchymal stem cells. Exp Mol Med.

[60] Le Bin GC (2014). Oct4 is required for lineage priming in the developing inner cell mass of the mouse blastocyst. Development.

[61] Niwa H, Miyazaki J, Smith AG (2000). Quantitative expression of Oct-3/4 defines differentiation, dedifferentiation or self-renewal of ES cells. Nat. Genet..

[62] Komori T (2010). Regulation of bone development and extracellular matrix protein genes by RUNX2. Cell Tissue Res..

[63] Kurata H, Guillot PV, Chan J, Fisk NM (2007). Osterix Induces Osteogenic Gene Expression but not Differentiation in Primary Human Fetal Mesenchymal Stem Cells. Tissue Engineering.

[64] About I (2000). Human dentin production *in vitro*. Exp. Cell Res..

[65] Pagella P, Neto E, Lamghari M, Mitsiadis TA (2015). Investigation of orofacial stem cell niches and their innervation through microfluidic devices. Eur Cell Mater.

[66] Chen S (2009). Runx2, osx, and dspp in tooth development. J. Dent. Res..

[67] Unda F-J (2000). Dissection of the odontoblast differentiation process *in vitro* by a combination of FGF1, FGF2, and TGFβ1. Developmental Dynamics.

[68] Gregoire FM (2001). Adipocyte differentiation: from fibroblast to endocrine cell. Exp. Biol. Med. (Maywood).

[69] Schindelin J (2012). Fiji: an open-source platform for biological-image analysis. Nat. Methods.

